# Predominance of *Blastocystis* sp. Infection among School Children in Peninsular Malaysia

**DOI:** 10.1371/journal.pone.0136709

**Published:** 2016-02-25

**Authors:** Kalimuthu Nithyamathi, Samudi Chandramathi, Suresh Kumar

**Affiliations:** 1 Department of Parasitology, Faculty of Medicine, University of Malaya, Kuala Lumpur, Malaysia; 2 Department of Medical Microbiology, Faculty of Medicine, University of Malaya, Kuala Lumpur, Malaysia; Royal Tropical Institute, NETHERLANDS

## Abstract

**Background:**

One of the largest cross-sectional study in recent years was carried out to investigate the prevalence of intestinal parasitic infections among urban and rural school children from five states namely Selangor, Perak, Pahang, Kedah and Johor in Peninsula Malaysia. This information would be vital for school authorities to influence strategies for providing better health especially in terms of reducing intestinal parasitism.

**Methods and Principal Findings:**

A total of 3776 stool cups was distributed to 26 schools throughout the country. 1760 (46.61%) responded. The overall prevalence of intestinal parasitic infection in both rural and urban areas was 13.3%, with *Blastocystis sp* (10.6%) being the most predominant, followed by *Trichuris trichiura* (3.4%), *Ascaris lumbricoides* (1.5%) and hook worm infection (0.9%). Only rural school children had helminthic infection. In general Perak had the highest infection (37.2%, total, n = 317), followed by Selangor (10.4%, total, n = 729), Pahang (8.6%, total, n = 221), Kedah (6.2%, total, n = 195) and Johor (3.4%, total, n = 298). School children from rural schools had higher infection (13.7%, total, n = 922) than urban school children (7.2%, total, n = 838). Subtype (ST) 3 (54.3%) is the most predominant ST with persons infected with only ST1 and ST3 showing symptoms. *Blastocystis* sp infection significantly associated with low household income, low parent’s education and presence of symptoms (p<0.05).

**Conclusion:**

It is critical that we institute deworming and treatment to eradicate the parasite especially in rural school children.

## Introduction

Intestinal parasitic infection (IPI) is common with an estimation of 3 billion people infected worldwide. It is a major public health problem in Southeast Asia particularly among poor children living in urban squats and rural communities. In Malaysia, intestinal parasitic infection is endemic among Orang Asli communities [1,2]. High infection rates are associated with high human population density, low socio-economic status, inadequate supplies of clean water, insanitary disposal of feces and larger families [2]. Infection distribution in a community follows a negative binomial pattern, although everybody is susceptible, most individuals are uninfected or have low infection intensity, whilst only a small proportion carry a heavy parasitic load [3].

Epidemiological studies carried out previously showed that the socioeconomic situation can be one of the major contributors to disease transmission caused by parasitic diseases. *Blastocystis* sp. have been shown to be the commonest intestinal parasite found in most stool surveys[4]. Most of the prevalence studies carried out in Malaysia focused on aborigines [1,5,6], HIV infected patients or immunocompromised patients[7], closed communities namely high-rise flat dwellers [8,9], patients diagnosed with gastrointestinal disorders such as Irritable Bowel Syndrome (IBS) [10] and colorectal cancer patients [11]. The cohort group never exceed sample of 300 except two studies which examined 500 stool samples form the aborigine community. There are increasing reports implicating that the parasite causes diarrhoea and stomach bloating [12–14]. The prevalence of *Blastocystis* sp. varies with different types of population however it can be seen that a large scale survey has never been carried out (Table 1). Furthermore, extensive studies on this parasite showed that *Blatocystis* sp. infection is not restricted to or just affecting developing countries such as Bangladesh [15], China [16], Nepal [17], Pakistan [15], Thailand [18], Turkey [19] but also developed countries such as Denmark [20], France [21], Germany [15], Japan [15], Singapore [22] and United States [4].

**Table 1:** Prevalence studies on Intestinal parasitic infection in Malaysia from 1999 to 2013.

No	Type of parasite	Type of population	Location	Total sample size	Year	Reference
1	Intestinal parasite	aborigine children	Kelantan	111	2013	[23]
2	*Blastocystis* sp.	Orang Asli	Negeri Sembilan, Perak and Pahang	500	2014	[24]
3	Giardia sp.	Orang Asli	Selangor, Perak and Pahang	500	2012	[25]
4	*Blastocystis* sp.	rural primary schoolchildren	Pahang	300	2012	[26]
5	Intestinal parasite	rural community	West Malaysia	550	2012	[27]
6	Intestinal parasite	HIV-infected individuals	Malaysia	346	2011	[28]
7	Cryptosporidium sp	Orang Asli	Selangor	276	2011	[29]
8	Giardia sp.	Orang Asli	Pahang	321	2008	[30]
9	soil-transmitted helminths	Orang Asli	Selangor	281	2007	[31]
10	Intestinal parasite	Orang Asli	Cameron highland	262	2007	[32]
11	intestinal protozoa	Orang Asli	Pahang	130	2007	[6]
12	Cryptosporidium sp.sp	HIV-infected	Kajang Hospital, Selangor	66	2005	[33]
13	Intestinal parasite	public	Kuala Lumpur	246	2005	[9]
14	Intestinal parasite	interior communities	Rejang River, Sarawak	355	2002	[34]
15	Intestinal parasite	aborigine children	Kelantan	162	1997	[35]
16	Giardia duodenalis	rural community	Malaysia	917	1998	[36]
17	*Blastocystis* sp.	animal handlers	local research institutions and zoo	105	1999	[37]

There has not been a pervasive prevalence study on IPI throughout the nation. As the nation is progressing towards a developed status, one of the indicators of achievement is health. Prevalence of parasitic infections in primary school children from rural and urban schools will provide health indicators. The questionnaire reflecting the association of these children to socio-demographic factors, environmental factors, behavioural habits and gastrointestinal complaints would further provide a gauge to assess health and the respective risks among children aged 5–12 years in the country.

## Materials and Methods

### Study area and population

**Fig 1:**
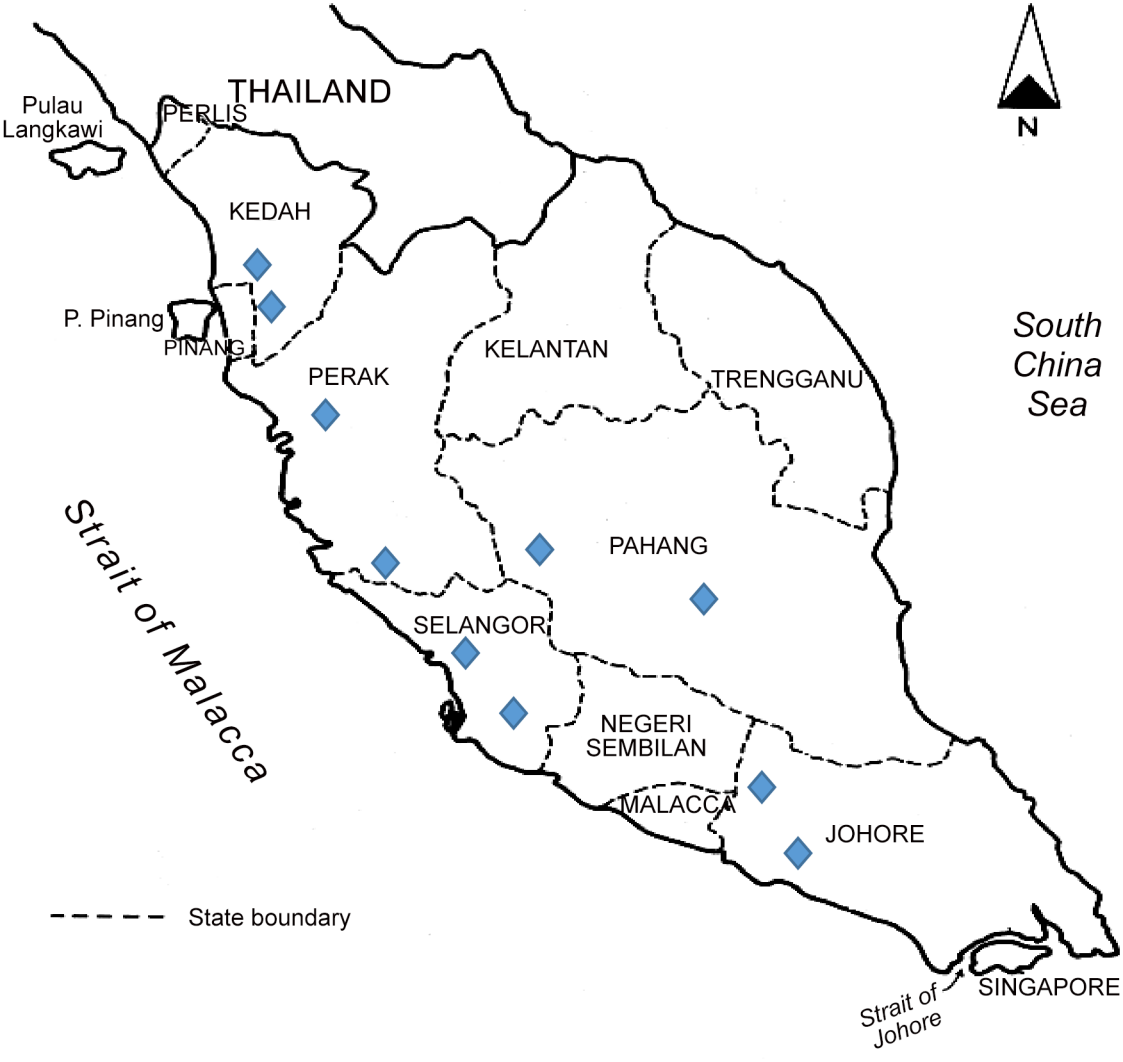
Geographical map of Peninsular Malaysia and study areas. —Indicates sample collection sites including both rural and urban areas. The rural and urban schools were identified based on the classification provided in the website of Ministry of Education, Malaysia.

A cross-sectional survey was carried out between May 2012 and October 2013 among primary school children (from age 7 to 12) from five different states of Peninsular Malaysia i.e.: Perak, Selangor, Johor, Pahang and Kedah (Fig 1). Kedah and Perak represent northern region, Pahang and Selangor represent central region and Johor represents southern region of Peninsular Malaysia This study was approved by National Ministry of Education and the respective State Departments of Education. Rural and urban schools were randomly selected based on the official list of schools published in the respective State Education’s web portal. According to Ministry of Rural and Regional Development, Malaysia, rural areas are referred to areas with population less than 10,000 people having agriculture and natural resources in which its population either clustered, linear or scattered. In contrast, urban areas are known as gazetted areas with population of 10 000 and more. The school administrators (i.e. headmaster, school teachers and staff) were informed about the objectives and procedures involved in the study. Literate parents or legal guardians of school children were informed and consent (both written and verbal) were obtained prior to the surveys being carried out. Participation was voluntary, and hence, children could withdraw at any time from the study without any obligations. The list of school children names, age, class and gender were obtained prior to sample collection in order to categorize them accordingly.

### Questionnaire

A structured questionnaire was prepared in English and Bahasa Malaysia (the national language of Malaysia). An oral briefing on IPI and hygiene practices to school children was given before the commencement of sample collection. The questionnaire was then distributed to children to be filled by their respective parents or guardian. school children were requested for demographic data (i.e., age, gender and education level), socioeconomic background of parents (household income and educational status) and also asked if they had any gastrointestinal symptoms or abnormalities (i.e. diarrhoea, abdominal pain or bloating, bloody or watery stools, constipation, vomiting).

### Faecal examination

Sterile stool containers were distributed to all the school childrenon the day before sample collection. All school children were briefed on the procedure to collect and handle faecal samples. Faecal examination was performed by adding approximately pea size of faecal sample into Jones’ medium supplemented with 10% horse serum, incubated at 37°C and examined using light microscope for the subsequent 48 to 72 hours. Direct faecal examination and formal ether concentration techniques were used to examine the presence of other intestinal parasites using light microscope.

### Subtyping of *Blastocystis* sp.

Faecal samples positive for *Blastocystis* sp. by *in vitro* cultivation in Jones’s medium and identified by light microscopy were subjected to DNA extraction using a commercial kit, QIAamp Deoxyribonucleic acid (DNA) Stool Mini Kit (Qiagen GmbH, Hilden, Germany) following the manufacturer’s instructions. Extracted DNA was stored at -20°C until used for genotyping of *Blastocystis* sp. isolates. The samples were subjected to polymerase chain reaction (PCR) amplification using sequenced tagged site (STS) primer with seven sets of primers (SB83, SB155, SB227, SB332, SB340, SB336 and SB337) for the genotyping of *Blastocystis* sp. from subtype 1 to subtype 7 (ST1 –ST7) (Yoshikawa et al. 2004). *Blastocystis* sp. ST1- ST7 are the most common subtypes that infect humans [21]. Therefore, the primer sequences specific for ST8 and ST9 were not used in the present study. Samples that were positive in *in vitro* cultivation but negative for subtyping classification using primer ST 1- ST 7 were labelled as unknown subtypes. The PCR conditions used for subtyping consisted of one initial denaturing cycle at 94°C for 3 min followed by 30 cycles that started with denaturing at 94°C for 30 seconds, annealing at 56.3°C for 30 seconds, extending at 72°C for 1 minute and an additional cycle of 10 minutes chain elongation at 72°C (Thermocycler, Bio-Rad). PCR products were separated in 1.5% agarose gel.

### Statistical analysis

Data entry was carried out in Microsoft Excel 2007 spreadsheet and statistical analyses were carried out in SPSS Statistics version 17.0 software. Descriptive chi square test as appropriate was used to assess the relationship of the prevalence of *Blastocystis* sp. infection between demographic factor (i.e age, gender), socioeconomically status and symptoms caused by infection. Univariate analysis were used to identify the potential risk factor between each variable while multivariate analysis employing forward logistic regression model was used to identify the significant predictors. The frequency of the various subtypes of *Blastocystis* sp. was also assessed.

### Ethical consideration

The study protocol was approved by the Ethics Committee of the University Malaya Medical Centre (UMMC), Malaysia (Reference Number: 848.28) prior to the commencement of the study. The participants were informed that the procedure used did not pose any potential risk and their identities and personal particulars would be kept strictly confidential.

## Results

The response from the respective five states (Perak, Selangor, Johor, Pahang and Kedah) is shown in Table 2. Although 3776 questionnaires and faecal containers were distributed during the study, only 1760 (46.61%) school children turned up with their respective faecal samples. These school children consisted of 937 (53.20%) male and 823(46.8%) female with ages between 7 to 9 years (604 (34.3%) and 10 to 12 years (1156 (65.7%) respectively. The demographic data including background information of school children such as age, gender, household income of family, and parents’ education level are shown (Table 3).

**Table 2:** The rate of response of school children from different states.

State	Rural		Urban	
	Distributed, N	Responded, N (%)	Distributed, N	Responded, N (%)
Perak	302	188 (62.25%)	301	129 (42.86%)
Selangor	766	349 (45.56%)	789	380 (48.16%)
Johor	340	148 (43.53%)	150	47 (31.33%)
Pahang	243	92 (37.86%)	225	129 (57.33%)
Kedah	329	145 (44.07%)	331	153 (46.22%)
Total	1760 / 3776 (46.61%)			

**Table 3:** The general characteristics of the school children such as household income of family, and parent’s education background.

Factors	Rural, n = 922	Urban, n = 838	Overall, n = 1760
*Gender*			
Male	463(50.2%)	474(56.6%)	937(53.20%)
Female	459(49.8%)	364(43.4%)	823(46.8%)
*Age group*			
Age (7–9)	320(34.7%)	284(33.9%)	604(34.3%)
Age (10–12)	602(65.3%)	554(66.1%)	1156(65.7%)
*Household Income*			
Low	438(47.5%)	147(17.5%)	585(33.2%)
Middle	419(45.4%)	487(58.1%)	906(51.5%)
High	64(6.9%)	204(24.3%)	268(15.2%)
*Father’s education level*			
Primary	214(23.2%)	25(3.0%)	239(13.7%)
secondary	673(73.0%)	662(79.0%)	1335(75.9%)
Tertiary	34(3.7%)	151(18%)	185(10.5%)
*Mother’s education level*			
Primary	257(27.9%)	34(4.1%)	291(16.5%)
secondary	638(69.2%)	710(84.7%)	1348(76.6%)
Tertiary	27(2.9%)	94(11.2%)	121(6.9%)

### Prevalence of Intestinal parasitic infection

The overall prevalence of intestinal parasite infection (IPI) among 1760 school children was 13.3% (n = 234), the parasites found were *Blastocystis* sp 10.6% (n = 186), *Trichuris trichiura* 3.4% (n = 59), *Ascaris lumbricoides* 1.5% (n = 26) and hookworm 0.9% (n = 16). *Blastocystis* sp. was found in both rural (13.70%; n = 922) and urban (3.40%; n = 838) school children. Whereas other parasites such as *Trichuris trichiura* (6.40%), *Ascaris lumbricoides* (2.80%), hookworm (1.70%) *Giardia* sp. (0.002%) and *Taenia* sp. (0.001%) can be found only in rural areas (n = 922) (Table 4). *Blastocystis* sp. is the most predominant parasite among all the parasites.

**Table 4:** Percentage of intestinal parasitic infection among school children from rural and urban in Peninsular Malaysia.

Name of intestinal	Percentage of positive sample		
parasite	Urban, n = 838	Rural, n = 922	Total, n = 1760
***Blastocystis* sp.**	3.40%	13.70%	10.6% (n = 186)
***Trichuris trichiura***	0%	6.40%	3.4% (n = 65)
***Ascaris lumbricoides***	0%	2.80%	1.5% (n = 26)
**Hookworm**	0%	1.70%	0.9% (n = 16)
**Giardia sp**	0%	0.002%	0.001% (n = 2)
**Taenia sp.**	0%	0.001%	0.0005% (n = 1)

### Prevalence of *Blastocystis* sp. infection

The overall prevalence of *Blastocystis* sp. infection was 10.6%. The highest prevalence rate was evident in the rural area (13.7%; n = 126) whereas the urban area had 7.2% (n = 60). In total, Perak showed highest prevalence rate with 24.0% followed by Selangor (9.5%), Pahang (8.6%), Kedah (6.2%) and Johor (3.4%) (Table 5). Subtype (ST) 3 (54.3%) had been reported to be the most predominant among all the subtypes, followed by ST 1 (22.6%), ST 2 (7.0%), ST 4 (7.0%) and ST 5(3.2%). There were no ST6 and ST7 found in these subjects (Table 6). In addition, 2.2% (4/186) were mixed infections consisting of two subtypes, which are ST1 and ST2 (n = 1) as well as ST1 and ST3 (n = 3). Unknown subtypes (negative for subtyping classification using primer ST1-ST7) also found in this study which is 1.6% (3/186). Generally, school children with ST3 and ST1 infection had symptoms such as diarrhoea, abdominal pain, abdominal bloating and constipation whereas other subtypes did not show any symptoms

**Table 5:** Prevalence of *Blastocystis* sp. infection among school children from different states, namely Selangor, Perak, Johor, Pahang and Kedah.

State	Rural	Urban	Total
**Selangor**	37/349 (**10.6%**)	32/380 (**8.2%**)	69/729(9.5%)
**Perak**	58/188 (**30.9%**)	18/129 (**14%**)	76/317(24.0%)
**Johor**	10/145 (**6.9%)**	0/153 (**0%**)	10/298(3.4%)
**Pahang**	11/92 (**12.1%**)	8/129 (**6.2%**)	19/221(8.6%)
**Kedah**	10/148 **(6.8%**)	2/47 (**4.3%**)	12/195(6.2%)
**Total**	**126 (13.7.7%)**	**60 (7.2%)**	**186/1760(10.6%)**

**Table 6:** Subtype classification from *Blastocystis* sp isolates from different states.

Area	No of *Blastocystis* sp.infection	ST1	ST2	ST3	ST4	ST5	UNKNOWN*/ Co-INFECTION^#^
Rural							
Selangor	37	10	3	22	1	1	0
Perak	58	12	7	32	5	2	0
Johor	10	2	0	5	2	0	1
Pahang	11	2	1	7	0	0	1
Kedah	10	4	0	4	1	0	1
Total	126	28(22.2%)	10(7.9%)	69(54.8%)	9(7.1%)	3(2.4%)	3(2.4%)
Urban							
Selangor	32	6	2	17	3	2	2
Perak	18	5	0	10	1	0	2
Johor	0	0	0	0	0	0	0
Pahang	8	3	1	3	0	1	0
Kedah	2	0	0	2	0	0	0
Total	60	14(23.3%)	3(5%)	32(53.3%)	4(6.7%)	3(5%)	4(6.7%)
Overall	186	42(22.6%)	13(7.0%)	101(54.3%)	13(7.0%)	6(3.2%)	7(3.8%)

* Unknown: samples are positive in *in vitro* cultivation but negative for subtyping classification using primer ST 1- ST 7.

^#^Co-infection: samples that are positive for more than one subtype.

### Risk factors of *Blastocystis* sp. infection

The risk factors associated with *Blastocystis* sp. infection in relation to demographic, geographical location and socioeconomic factors among rural and urban communities were examined using univariate analysis. The results showed that demographic factors of the school children age (7–9 years old (10.3%) vs 10–12 years old (10.70%), p = 0.807) and gender (male (12.0%) vs female (9.0%), p = 0.052) were not significantly associated with *Blastocystis* sp. infection. Risk factors were identified which include family’s low household income (OR = 4.223, 95% CI = 3.072–5.806; p<0.001), fathers’ low education level (OR = 5.321, 95% CI = 3.803–7.445; p<0.001), mothers’ low education level (OR = 5.011, 95% CI = 3.623–6.929; p<0.001), presence of symptoms (OR = 5.963, 95% CI = 4.304–8.261; p<0.001) and rural area (OR = 2.053, 95% CI = 1.486–2.835; p<0.001). (Table 7) Multivariate analysis using forward logistic regression model further confirmed that school childrenwith low household income had 2 times (95% CI = 1.279–2.869, p<0.001) and presence of symptoms had 6 times (95% CI = 4.281–8.838, p<0.001) possibilities of suffering from *Blastocystis* sp. infections, respectively.

**Table 7:** Potential risk factor associated with *Blastocystis* sp. infection (Univariate analysis, n = 1760).

Variables	N	No	%	OR (95%CI)	*p* value
*Gender*					
Male	937	112	12.00%	1.374(.008–1.873)	0.052
Female	823	74	9.00%	1	
*Age group*					
Age (7–9)	604	62	10.30%	0.952(0.690-.1.314)	0.807
Age (10–12)	1156	124	10.70%	1	
*Household Income*					
<2000	585	119	20.30%	4.223(3.072–5.806)	<0.001**
>2000	1175	67	5.70%	1	
*Presence of symptoms*					
Symptomatic	254	83	32.70%	5.963(4.304–8.261)	<0.001**
Asymptomatic	1506	103	6.80%	1	
*Education Of father*					
Primary	239	72	30.10%	5.321(3.803–7.445)	<0.001*
Secondary	1521	114	7.50%	1	
*Education of Mother*					
Primary	291	81	27.80%	5.011(3.623–6.929)	<0.001*
Secondary	1469	105	7.10%	1	
*Area*					
Rural	922	126	16.40%	5.391(3.564–8.154)	<0.001
Urban	838	60	7.20%	1	

N: Number examined; no: Number positive.

Reference group marked as OR = 1; CI: Confidence interval.

*Significant association (p<0.05).

**Variables were confirmed by multivariate analysis as significant predictors of *Blastocystis* infection.

## Discussion

To the best of our knowledge, this is the first large scale study done in Peninsular Malaysia to provide a prevalence data on intestinal parasitic infection namely *Blastocystis* sp. among urban and school children. In present study, the prevalence of *Blastocystis* sp. were determined based on *in vitro* culture technique. Generally, *in vitro* culture technique is used for routine laboratory screening for the rapid detection of *Blastocystis* sp as it is reported *in vitro* culture using Jones' medium is more sensitive than the formalin-ether concentration technique for detecting *B*. *hominis* [38] We agree that other molecular techniques namely PCR [20] and qPCR [39] which have been reported to be more sensitive for *Blastocystis* sp. detection could have increased the prevalence rate in this study. However, in our study the *in vitro* technique would be the best choice as it is more economical in screening large sample size.

The results showed that out of 1760school children, 234 (13.3%) were infected with at least one type of intestinal parasite with infection rate of 18.9% and 7.2% in rural and urban area respectively. The data showed that intestinal parasitic infection (IPI) is one of the major public health problems among underprivileged and socioeconomically deprived communities in developing countries such as Malaysia. Our study also showed that IPI is highly prevalent in Perak (24%). This is due to the fact that majority of school children of this state were from the aborigine community and had low household income compared to other states. Many previous studies reported that aborigine communities had high IPI as a consequence of their poor socio-economic environment.

The present study revealed high prevalence of intestinal protozoa infection namely *Blastocytis sp*. (13.70%) followed by Soil Transmitted Helminths (STH) such as *Trichuris trichiura* (6.4%), *Ascaris lumbricoides* (2.8%) and hookworm (1.7%) among school children from rural area. According to a previous study, prevalence of STH infection was at 41.4% among Malaysian children (from 0 to 15 years old). The present study showed that the prevalence of STH had decreased compared to previous study [40]. The present study showed that *Giardia* sp. (0.002%) and *Taenia* sp. (0.001%) were present but had the least infection rate. Between year 1992 and 1994 a study carried out to determine the prevalence of giardiasis among Malaysian primary school children (n = 7557) from lower socio-economic group showed 0.21% [41]. The present study showed that there is almost a 100-fold reduction in the infection rate of *Giardia* sp. in the past 10 years and this can be considered as a reflection of development especially in terms of proper toilet facilities and sanitation, water supply and hygiene practice in Malaysia.

To date, most of the studies reported on IPI among Malaysian school children had focused only on aborigine communities in selected areas [1,6,26] (Table 1) and the high prevalence in these communities was associated with low socioeconomic status. A recent report showed that the IPI was present in 52.3% among the aborigine community in Pahang [6] and 20.7% among three different types of Orang Asli tribes [27]. The present study is the first to compare IPI among both urban and rural school children in Malaysia. In this study we found transmission of IPI only prevalent in rural schools. This cannot be obviously due to poor sanitary and contaminated water supplies as development has reached most parts of the rural areas. It is highly possible that playing in fields, bare-footed, contaminated with STH as well as low public health awareness may contribute to the transmission. However in the case of *Blastocystis* sp. the organism reflected highest prevalence (10.6%), which is evident in both rural (13.7%) and urban (7.2%) school children. In the past, prevalence of *Blastocystis* sp. in Malaysia only focused on cohort groups of children in aborigines or rural communities (25.7%) [26], in cancer patients (4.0%) [42] and patients with diarrhoea (4.4%) [1]. The only other study showing a prevalence in urban population was the report of 10.2% in high rise flat dwellers in Kuala Lumpur [8]. In addition to that, epidemiological studies [43,44] also provided molecular evidence supporting possible human-to-human and waterborne zoonoses of *Blastocystis* sp. within communities living in close proximity with animals and in the vicinity of water sources.

In a previous study, 615 water samples were collected from May 2008 till April 2010 including bottled drinking water, filtered water, lakes, ponds, rivers, tap and well water in Peninsular Malaysia and 11.7% of samples were contaminated with *Blastocystis* sp. (unpublished work). This study also showed that *Blastocystis* sp. was detected in 37.0% of river water samples from Selangor state and Federal Territory of Kuala Lumpur where most of the rivers were surrounded with housing and commercial properties which may potentially contribute to the contamination. Accidental consumption of recreational river water contaminated with *Blastocystis* sp. by visitors may be a cause of transmission. The present study shows almost a similar prevalence of *Blastocystis* sp. in rural (10.6%) and urban schools (8.2%) in the state of Selangor. The statistics reveal that despite changing landscape in these two settings similar transmission patterns appear to be going on. Moreover the high population influx of people from other states to Selangor state seeking study and employment opportunities could be a contributory factor.

*Blastocystis* sp. is classified into 17 subtypes (ST1- ST17) based on nomenclature established by Stensvold et al. [45]. In the current study, *Blastocystis* sp. ST3 had the highest prevalence (54.3%) followed by ST1 (22.6%), ST2 (7.0%), ST4 (7.0%) and ST5 (3.2%). ST 1 –ST 4 are common among humans of which ST3 being the most predominant subtype. ST5—ST9 have been sporadically isolated from humans while ST10 –ST17 have not been found in humans [17]. In Asian countries such as Bangladesh [15], China [15], Iran [46], Japan [15], Malaysia [47], Pakistan [48], Singapore [22] and Thailand [18,49], ST1 as well as ST3 are the most predominant subtypes.

The epidemiological characteristics such as reservoir and transmission methods imply the variation of subtypes in different locations. In relation to this, *Blastocystis* sp. was also detected in drinking water[26], sewage[50] and rivers from recreational areas [51,52] in Malaysia. The World Health Organisation publications on drinking water quality have included *Blastocystis* sp. as one of the pathogens to be considered for waterborne zoonosis and thus providing evidence for waterborne transmission by this parasite [26]. Children in the rural schools may come from families with agricultural and farming background. This can also be a route of acquiring the infection as it was shown previously that *Blastocystis* was highly prevalent among animal handlers (41%) [37]. A study pertaining to zoonotic transmission with molecular evidence has shown the transmission of this parasite from animals to animal handlers in Philippines and Australia [53,54]. Besides that, *Blastocystis* sp. ST2 was detected in children and monkeys living within the same area in Kathmandu, Nepal and presence of *Blastocystis* sp. subtype 4 was seen in those who were raring animals next to their dwellings [17]

Subsequently, risk factors such as age, gender were analysed using univariate and multivariate analysis to identify the predictors of *Blastocystis* sp. infection among children in Malaysia. Based on our result, age did not show any significant value. This shows that there was no difference in socio behaviour between age groups 7–9 (10.3%) and 10–12 (10.7%) though the infection rates of children aged 10–12 was slightly higher than aged 7–9. With regards to gender, *Blastocystis* sp infection rate among male (12.0%) is slightly higher than female (9.0%) and shows marginally significant (p = 0.052). In previous studies, *Blastocystis* sp. infection were associated with numerous factors such as consumption of contaminated food and water, close contact with animals, poor personal hygiene, inadequate sanitation, geographical distribution and seasonal influences [4,37,55].

Our findings confirmed that, family’s low household income especially in rural communities and presence of symptoms were significant risk predictors of IPIs namely *Blastocystis* sp. infection. Previous studies substantiate that poor and socioeconomically deprived rural communities and the poor people of under developed nations are highly possible to acquire the infection due to active transmission within the community [2,27]. Health education and sanitation are two important aspects of primary health care system introduced by the World Health Organization (WHO) as a basis for the prevention and control of communicable diseases. Lower level of education in the present study possess a risk as parents due to lack of awareness on hygiene practices may play a major role in facilitating a transmission.

Besides that, *Blastocystis* sp. infection was found to be significantly associated with gastrointestinal symptoms among these school children. Interestingly in present study, we found that isolates from symptomatic participants belonged to ST1 and ST3, whereas ST2, ST4 and ST5 were isolated from asymptomatic participants. There was a significant difference in the distribution of subtypes between the symptomatic and asymptomatic groups (p<0.001)., A previous study did show the association of ST1 and ST3 with pathogenicity [56]. Furthermore, a study in Denmark showed that most of the patients with suspected parasitic gastrointestinal infection were positive for *Blastocystis* sp. ST1 and ST3 [20]. In addition, previous studies revealed that ST3 and ST1 respectively correlates with gastrointestinal symptoms [57] [58]. Our study concur with the previous findings that the pathogenic potential of this parasite is attributed to subtype variation. [59].

Although in early years some clinical studies concluded that *Blastocystis* sp is a commensal and may not be responsible for clinical symptoms [60], recently, several epidemiological studies have reported on the positive correlation between gastrointestinal symptoms with *Blastocystis* sp. Infection [13, 56]. Sheehan et al. [61] had reported that a total of five or more *B*. *hominis* cells per 40x magnification field seen in a direct microscopic examination of stool smear wet mount is suggestive of a pathogen that causes clinical illnesses. A recent study conducted on patients with confirmed positive *Blastocystis* sp. and aimed to assess the frequency, clinical symptoms and skin manifestations, found that 73.75% of the patients had gastrointestinal symptoms[62]. In another study conducted among Malaysian primary rural school children revealed that infection of *Blastocystis* sp. regardless of single or multiple infection was found to be significantly associated with symptoms such as abdominal pain (58.8%) and diarrhoea (50.0%) [26]. On the other hand, predominance of amoeboid forms of *B*. *hominis* in isolates from symptomatic patients[63] had been demonstrated and this amoebic forms of *Blastocystis* sp. could play a major role in exacerbating the gastrointestinal symptoms[64]

## Conclusion

Intestinal parasitic infections namely *Blastocystis* sp. are highly prevalent among school children from poor and socioeconomically deprived rural communities in Peninsular Malaysia. Our findings may serve as baseline data to the government authorities in order to eradicate intestinal parasitic infection such as STHs and protozoan infection. There is a need to conduct screening of IPIs namely for *Blastocystis* sp. among school children as it may suppress the immune system and subsequently leading to secondary infectious diseases. Therefore, in order to control the infection, it is necessary to promote health awareness and hygiene practices especially in school children.
